# 1,4-Diazo­niabicyclo­[2.2.2]octane bis­(2-chloro­benzoate)

**DOI:** 10.1107/S1600536808020096

**Published:** 2008-07-05

**Authors:** Signe Skovsgaard, Andrew D. Bond

**Affiliations:** a Department of Physics and Chemistry, University of Southern Denmark, Campusvej 55, 5230 Odense, Denmark

## Abstract

The title compound, C_6_H_14_N_2_^2+^·2C_7_H_4_ClO_2_^−^, contains trimeric units linked by N—H⋯O hydrogen bonds. The carboxyl­ate groups of the 2-chloro­benzoate anions form dihedral angles of 66.1 (1) and 76.1 (1)° with the respective chloro­benzene rings to which they are bound. The hydrogen-bonded trimers are arranged in layers in the (200) planes and the chloro­benzoate anions form edge-to-face inter­actions between layers, with dihedral angles of 61.9 (1) and 49.8 (1)° and centroid–centroid distances of 4.85 (1) and 4.65 (1) Å, respectively, for two crystallographically distinct inter­actions.

## Related literature

For other co-crystals of 1,4-diazo­niabicyclo­[2.2.2]octane and carboxylic acids, see: Meehan *et al.* (1997 ▶); Burchell *et al.* (2000 ▶); Burchell, Glidewell *et al.* (2001 ▶); Burchell, Ferguson *et al.* (2001 ▶). For the crystal structure of 2-chloro­benzoic acid, see: Ferguson & Sim (1961 ▶).
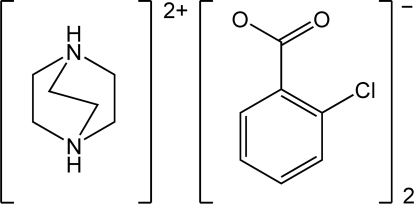


## Experimental

### 

#### Crystal data

C_6_H_14_N_2_^2+^·2C_7_H_4_ClO_2_^−^
*M* *_r_* = 425.30
Orthorhombic, 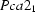

*a* = 19.7694 (12) Å
*b* = 11.3986 (6) Å
*c* = 8.9751 (5) Å
*V* = 2022.5 (2) Å^3^
*Z* = 4
Mo *K*α radiation
μ = 0.35 mm^−1^
*T* = 298 (2) K
0.30 × 0.20 × 0.10 mm

#### Data collection

Bruker–Nonius X8 APEXII CCD diffractometer
Absorption correction: multi-scan (*SADABS*; Sheldrick, 2003 ▶) *T*_min_ = 0.844, *T*_max_ = 0.966
21779 measured reflections
3538 independent reflections
3251 reflections with *I* > 2σ(*I*)
*R*_int_ = 0.023

#### Refinement

*R*[*F* ^2^ > 2σ(*F* ^2^)] = 0.033
*wR*(*F* ^2^) = 0.090
*S* = 1.06
3538 reflections
253 parameters
1 restraint
H-atom parameters constrained
Δρ_max_ = 0.26 e Å^−3^
Δρ_min_ = −0.19 e Å^−3^
Absolute structure: Flack (1983 ▶), 1629 Friedel pairs
Flack parameter: −0.02 (5)

### 

Data collection: *APEX2* (Bruker, 2004 ▶); cell refinement: *SAINT* (Bruker, 2003 ▶); data reduction: *SAINT*; program(s) used to solve structure: *SHELXTL* (Sheldrick, 2008 ▶); program(s) used to refine structure: *SHELXTL*; molecular graphics: *SHELXTL*; software used to prepare material for publication: *SHELXTL*.

## Supplementary Material

Crystal structure: contains datablocks global, I. DOI: 10.1107/S1600536808020096/bx2155sup1.cif

Structure factors: contains datablocks I. DOI: 10.1107/S1600536808020096/bx2155Isup2.hkl

Additional supplementary materials:  crystallographic information; 3D view; checkCIF report

## Figures and Tables

**Table 1:** Hydrogen-bond geometry (Å, °)

*D*—H⋯*A*	*D*—H	H⋯*A*	*D*⋯*A*	*D*—H⋯*A*
N1—H1*A*⋯O1	0.91	1.65	2.556 (2)	170
N2—H2*A*⋯O3	0.91	1.69	2.587 (2)	169

**Fig. 1.**
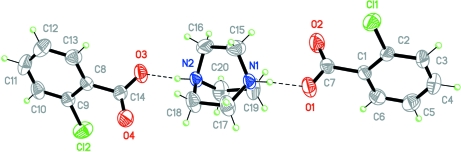
Molecular structure of the title compound showing displacement ellipsoids at the 50% probability level for non-H atoms. The dashed lines denote N+—H···O- hydrogen bonds.

**Fig. 2.**
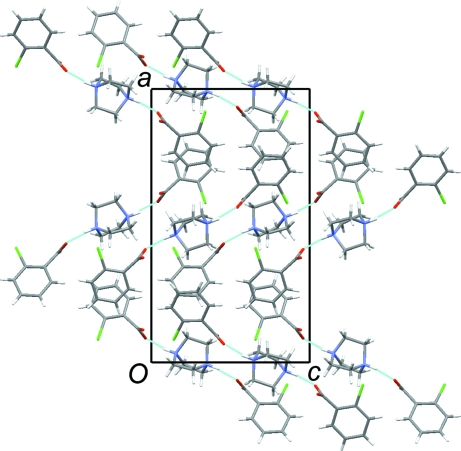
View along the c axis, showing the layered arrangement of hydrogen-bonded trimers. The light blue lines denote N+—H···O- hydrogen bonds.

**Fig. 3.**
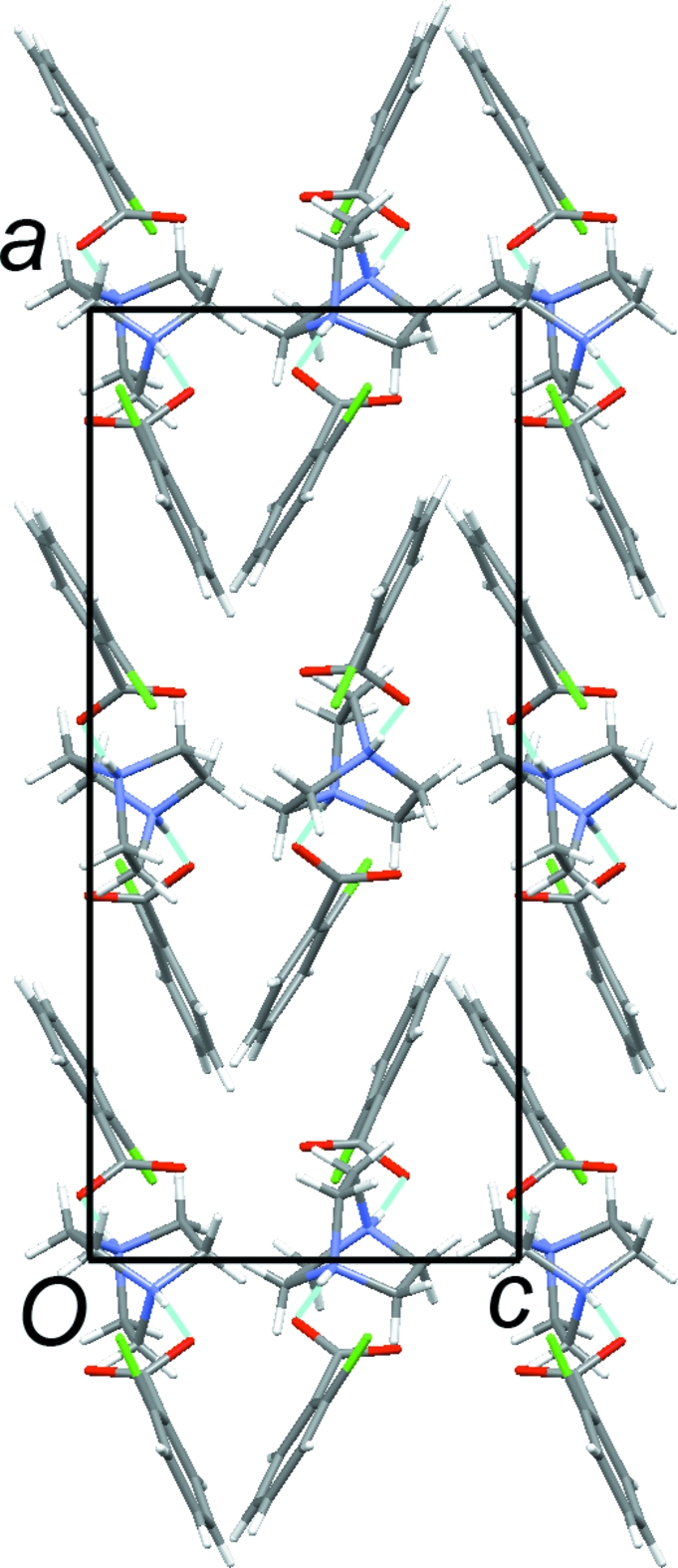
View along the b axis. The light blue lines denote N+—H···O- hydrogen bonds.

## supplementary crystallographic information

### Comment

The title compound, (C_6_H_14_N_2_)(C_7_H_4_ClO_2_)_2_, was obtained by co-crystallization of diazabicylo[2.2.2]octane and 2-chlorobenzoic acid in methanol solution. The crystal structure of 2-chlorobenzoic acid (Ferguson & Sim, 1961) contains dimers formed by hydrogen bonds between the carboxyl groups. The purpose of the co-crystallization was to insert DABCO into the hydrogen-bonded dimer, to examine the influence on the intermolecular interactions between the 2-chlorobenzoic acid molecules.

The co-crystal contains the anticipated trimeric hydrogen-bond motif, with the trimers lying in layers parallel to the *bc* planes (Figs. 2 & 3). The Cl-substituents of the chlorobenzoate anions point into the centres of the layers, and the interlayer interactions comprise edge-to-face interactions involving H4A and H5A and their counterparts H11A and H12A, with dihedral angles 61.9 (1) and 49.8 (1)° and centroid-centroid distances 4.85 (1) and 4.65 (1) Å, for the two interactions respectively. The interactions are significantly different from the interlayer interactions in 2-chlorobenzoic acid itself, where adjacent rings form a dihedral angle of 49.3 (1)°, but the Cl-substituent points towards the adjacent ring centroid.

### Experimental

Separate saturated solutions of 2-chlorobenzoic acid (0.391 g, 0.0025 mmol) and diazabicyclo[2.2.2]octane (0.135 g, 0.0012 mmol) in warm methanol were combined and refluxed with stirring for 1 h. The solution was cooled slowly, giving colourless crystals of the title compound after *ca* 1 h.

### Refinement

H atoms bound to C atoms were placed geometrically and allowed to ride during refinement with C—H = 0.93–0.97 Å and *U*_iso_(H) = 1.2*U*_eq_(C). The H atoms bound to N1 and N2 were visible in a difference Fourier map, but were placed geometrically (N—H = 0.91 Å) and allowed to ride with *U*_iso_(H) = 1.2*U*_eq_(N). The assignment as a salt is consistent with expectations from p*K*_a_ values.

### Crystal data

**Table:**

C_6_H_14_N_2_^2+^·2C_7_H_4_ClO_2_^–^	*F*_000_ = 888
*M**_r_* = 425.30	*D*_x_ = 1.397 Mg m^−^^3^
Orthorhombic, *P**c**a*2_1_	Mo *K*α radiation λ = 0.71073 Å
Hall symbol: P 2c -2ac	Cell parameters from 9140 reflections
*a* = 19.7694 (12) Å	θ = 3.1–24.3º
*b* = 11.3986 (6) Å	µ = 0.35 mm^−^^1^
*c* = 8.9751 (5) Å	*T* = 298 (2) K
*V* = 2022.5 (2) Å^3^	Block, colourless
*Z* = 4	0.30 × 0.20 × 0.10 mm

### Data collection

**Table:**

Bruker–Nonius X8 APEXII CCD diffractometer	3538 independent reflections
Radiation source: fine-focus sealed tube	3251 reflections with *I* > 2σ(*I*)
Monochromator: graphite	*R*_int_ = 0.023
*T* = 298(2) K	θ_max_ = 25.0º
Thin–slice ω and φ scans	θ_min_ = 3.6º
Absorption correction: multi-scan(SADABS; Sheldrick, 2003)	*h* = −23→23
*T*_min_ = 0.844, *T*_max_ = 0.966	*k* = −13→12
21779 measured reflections	*l* = −10→10

### Refinement

**Table:**

Refinement on *F*^2^	Hydrogen site location: inferred from neighbouring sites
Least-squares matrix: full	H-atom parameters constrained
*R*[*F*^2^ > 2σ(*F*^2^)] = 0.033	*w* = 1/[σ^2^(*F*_o_^2^) + (0.0543*P*)^2^ + 0.354*P*] where *P* = (*F*_o_^2^ + 2*F*_c_^2^)/3
*wR*(*F*^2^) = 0.090	(Δ/σ)_max_ = 0.001
*S* = 1.07	Δρ_max_ = 0.26 e Å^−^^3^
3538 reflections	Δρ_min_ = −0.19 e Å^−^^3^
253 parameters	Extinction correction: none
1 restraint	Absolute structure: Flack (1983), 1629 Friedel pairs
Primary atom site location: structure-invariant direct methods	Flack parameter: −0.02 (5)
Secondary atom site location: difference Fourier map	

### Special details

**Table:**

Geometry. All e.s.d.'s (except the e.s.d. in the dihedral angle between two l.s. planes) are estimated using the full covariance matrix. The cell e.s.d.'s are taken into account individually in the estimation of e.s.d.'s in distances, angles and torsion angles; correlations between e.s.d.'s in cell parameters are only used when they are defined by crystal symmetry. An approximate (isotropic) treatment of cell e.s.d.'s is used for estimating e.s.d.'s involving l.s. planes.
Refinement. Refinement of *F*^2^ against ALL reflections. The weighted *R*-factor *wR* and goodness of fit *S* are based on *F*^2^, conventional *R*-factors *R* are based on *F*, with *F* set to zero for negative *F*^2^. The threshold expression of *F*^2^ > σ(*F*^2^) is used only for calculating *R*-factors(gt) *etc*. and is not relevant to the choice of reflections for refinement. *R*-factors based on *F*^2^ are statistically about twice as large as those based on *F*, and *R*- factors based on ALL data will be even larger.

### Fractional atomic coordinates and isotropic or equivalent isotropic displacement parameters (Å^2^)

**Table:**

	*x*	*y*	*z*	*U*_iso_*/*U*_eq_	
Cl1	0.58385 (3)	1.34004 (5)	0.57363 (7)	0.05244 (17)	
Cl2	0.41713 (4)	0.14065 (6)	0.64801 (8)	0.0658 (2)	
O1	0.58704 (9)	1.03874 (14)	0.7359 (2)	0.0556 (5)	
O2	0.61776 (12)	1.05458 (17)	0.5009 (2)	0.0716 (6)	
O3	0.43357 (9)	0.45727 (15)	0.4822 (2)	0.0542 (4)	
O4	0.40470 (10)	0.42055 (18)	0.7161 (2)	0.0653 (5)	
N1	0.53241 (8)	0.84726 (14)	0.6495 (2)	0.0351 (4)	
H1A	0.5474	0.9192	0.6783	0.042*	
N2	0.49115 (9)	0.64905 (14)	0.5697 (2)	0.0383 (4)	
H2A	0.4760	0.5771	0.5410	0.046*	
C1	0.67102 (10)	1.17744 (18)	0.6779 (2)	0.0357 (4)	
C2	0.66021 (10)	1.29512 (17)	0.6508 (2)	0.0353 (4)	
C3	0.70790 (11)	1.3795 (2)	0.6867 (3)	0.0459 (5)	
H3A	0.6996	1.4582	0.6666	0.055*	
C4	0.76781 (12)	1.3457 (2)	0.7524 (3)	0.0574 (7)	
H4A	0.8003	1.4019	0.7756	0.069*	
C5	0.77993 (13)	1.2290 (3)	0.7838 (3)	0.0590 (7)	
H5A	0.8200	1.2061	0.8299	0.071*	
C6	0.73135 (12)	1.1464 (2)	0.7456 (3)	0.0503 (6)	
H6A	0.7396	1.0677	0.7662	0.060*	
C7	0.62150 (12)	1.08377 (18)	0.6306 (3)	0.0402 (5)	
C8	0.34750 (10)	0.31988 (18)	0.5241 (2)	0.0349 (4)	
C9	0.34941 (10)	0.20060 (18)	0.5500 (2)	0.0389 (5)	
C10	0.30020 (12)	0.1256 (2)	0.4960 (3)	0.0506 (6)	
H10A	0.3033	0.0453	0.5132	0.061*	
C11	0.24651 (13)	0.1707 (2)	0.4167 (3)	0.0581 (7)	
H11A	0.2130	0.1209	0.3808	0.070*	
C12	0.24255 (13)	0.2890 (3)	0.3909 (3)	0.0582 (6)	
H12A	0.2061	0.3197	0.3381	0.070*	
C13	0.29285 (12)	0.3629 (2)	0.4434 (3)	0.0480 (6)	
H13A	0.2900	0.4429	0.4243	0.058*	
C14	0.39949 (10)	0.40479 (18)	0.5813 (3)	0.0390 (5)	
C15	0.49080 (17)	0.8594 (2)	0.5144 (3)	0.0643 (7)	
H15A	0.5158	0.9018	0.4387	0.077*	
H15B	0.4501	0.9033	0.5372	0.077*	
C16	0.47206 (16)	0.7375 (2)	0.4566 (3)	0.0635 (8)	
H16A	0.4238	0.7335	0.4375	0.076*	
H16B	0.4957	0.7219	0.3640	0.076*	
C17	0.49289 (16)	0.7947 (2)	0.7703 (3)	0.0612 (7)	
H17A	0.4571	0.8479	0.8003	0.073*	
H17B	0.5218	0.7807	0.8557	0.073*	
C18	0.46232 (14)	0.6791 (2)	0.7171 (3)	0.0584 (7)	
H18A	0.4724	0.6173	0.7881	0.070*	
H18B	0.4136	0.6865	0.7095	0.070*	
C19	0.59035 (12)	0.7722 (2)	0.6143 (4)	0.0605 (7)	
H19A	0.6217	0.7717	0.6974	0.073*	
H19B	0.6139	0.8028	0.5278	0.073*	
C20	0.56618 (12)	0.6470 (2)	0.5826 (4)	0.0594 (7)	
H20A	0.5861	0.6185	0.4906	0.071*	
H20B	0.5798	0.5951	0.6628	0.071*	

### Atomic displacement parameters (Å^2^)

**Table:**

	*U*^11^	*U*^22^	*U*^33^	*U*^12^	*U*^13^	*U*^23^
Cl1	0.0553 (3)	0.0404 (3)	0.0615 (4)	0.0067 (2)	−0.0201 (3)	−0.0033 (3)
Cl2	0.0831 (5)	0.0419 (3)	0.0725 (5)	0.0129 (3)	−0.0271 (4)	−0.0006 (3)
O1	0.0751 (11)	0.0435 (10)	0.0480 (10)	−0.0240 (8)	0.0158 (8)	−0.0078 (8)
O2	0.1056 (15)	0.0635 (12)	0.0456 (10)	−0.0416 (11)	0.0024 (10)	−0.0066 (9)
O3	0.0660 (10)	0.0450 (10)	0.0518 (10)	−0.0245 (8)	0.0108 (8)	−0.0083 (8)
O4	0.0916 (14)	0.0583 (12)	0.0459 (10)	−0.0319 (10)	−0.0071 (10)	−0.0053 (9)
N1	0.0452 (10)	0.0237 (9)	0.0365 (9)	−0.0071 (6)	0.0028 (8)	−0.0031 (8)
N2	0.0507 (10)	0.0275 (9)	0.0367 (9)	−0.0115 (7)	0.0034 (9)	−0.0037 (8)
C1	0.0422 (11)	0.0312 (10)	0.0339 (10)	−0.0038 (8)	0.0013 (8)	0.0011 (9)
C2	0.0407 (11)	0.0314 (11)	0.0339 (10)	−0.0006 (8)	−0.0050 (9)	−0.0029 (9)
C3	0.0535 (13)	0.0293 (11)	0.0548 (14)	−0.0077 (9)	−0.0042 (11)	−0.0029 (10)
C4	0.0486 (14)	0.0515 (16)	0.0722 (18)	−0.0153 (11)	−0.0054 (13)	−0.0106 (13)
C5	0.0471 (13)	0.0652 (18)	0.0646 (16)	0.0005 (12)	−0.0138 (12)	0.0029 (14)
C6	0.0558 (14)	0.0389 (13)	0.0563 (15)	0.0030 (10)	−0.0084 (12)	0.0079 (11)
C7	0.0549 (12)	0.0247 (11)	0.0410 (12)	−0.0012 (9)	−0.0017 (10)	−0.0003 (9)
C8	0.0369 (10)	0.0334 (11)	0.0344 (10)	−0.0019 (8)	0.0037 (8)	0.0010 (9)
C9	0.0423 (11)	0.0347 (11)	0.0397 (12)	−0.0015 (9)	0.0020 (9)	−0.0046 (10)
C10	0.0596 (15)	0.0373 (12)	0.0549 (14)	−0.0141 (11)	0.0100 (12)	−0.0085 (11)
C11	0.0412 (12)	0.0677 (17)	0.0655 (16)	−0.0144 (14)	−0.0008 (12)	−0.0238 (14)
C12	0.0422 (12)	0.0730 (18)	0.0593 (14)	0.0050 (14)	−0.0099 (11)	−0.0093 (15)
C13	0.0540 (13)	0.0431 (14)	0.0468 (13)	0.0025 (11)	−0.0040 (11)	0.0005 (10)
C14	0.0458 (11)	0.0274 (11)	0.0438 (12)	−0.0015 (9)	−0.0034 (11)	−0.0028 (10)
C15	0.101 (2)	0.0358 (14)	0.0562 (15)	0.0020 (14)	−0.0241 (15)	0.0025 (12)
C16	0.091 (2)	0.0486 (16)	0.0506 (13)	−0.0154 (14)	−0.0283 (14)	0.0028 (12)
C17	0.0835 (18)	0.0515 (15)	0.0488 (13)	−0.0258 (14)	0.0192 (13)	−0.0146 (12)
C18	0.0752 (18)	0.0469 (15)	0.0530 (14)	−0.0230 (13)	0.0240 (13)	−0.0150 (12)
C19	0.0451 (13)	0.0421 (14)	0.094 (2)	−0.0066 (10)	0.0046 (12)	−0.0102 (14)
C20	0.0556 (14)	0.0427 (14)	0.0798 (17)	0.0033 (11)	0.0064 (15)	−0.0115 (14)

### Geometric parameters (Å, °)

**Table:**

Cl1—C2	1.738 (2)	C8—C13	1.390 (3)
Cl2—C9	1.742 (2)	C8—C14	1.502 (3)
O1—C7	1.273 (3)	C9—C10	1.383 (3)
O2—C7	1.213 (3)	C10—C11	1.378 (4)
O3—C14	1.266 (3)	C10—H10A	0.930
O4—C14	1.228 (3)	C11—C12	1.370 (4)
N1—C17	1.464 (3)	C11—H11A	0.930
N1—C19	1.465 (3)	C12—C13	1.386 (4)
N1—C15	1.472 (3)	C12—H12A	0.930
N1—H1A	0.910	C13—H13A	0.930
N2—C16	1.480 (3)	C15—C16	1.529 (4)
N2—C18	1.480 (3)	C15—H15A	0.970
N2—C20	1.488 (3)	C15—H15B	0.970
N2—H2A	0.910	C16—H16A	0.970
C1—C2	1.380 (3)	C16—H16B	0.970
C1—C6	1.385 (3)	C17—C18	1.526 (3)
C1—C7	1.509 (3)	C17—H17A	0.970
C2—C3	1.385 (3)	C17—H17B	0.970
C3—C4	1.378 (4)	C18—H18A	0.970
C3—H3A	0.930	C18—H18B	0.970
C4—C5	1.381 (4)	C19—C20	1.531 (3)
C4—H4A	0.930	C19—H19A	0.970
C5—C6	1.388 (4)	C19—H19B	0.970
C5—H5A	0.930	C20—H20A	0.970
C6—H6A	0.930	C20—H20B	0.970
C8—C9	1.380 (3)		
			
C17—N1—C19	109.7 (2)	C11—C12—C13	119.9 (2)
C17—N1—C15	110.5 (2)	C11—C12—H12A	120.0
C19—N1—C15	108.3 (2)	C13—C12—H12A	120.0
C17—N1—H1A	109.4	C12—C13—C8	121.4 (2)
C19—N1—H1A	109.4	C12—C13—H13A	119.3
C15—N1—H1A	109.4	C8—C13—H13A	119.3
C16—N2—C18	110.9 (2)	O4—C14—O3	125.4 (2)
C16—N2—C20	108.5 (2)	O4—C14—C8	119.2 (2)
C18—N2—C20	108.6 (2)	O3—C14—C8	115.4 (2)
C16—N2—H2A	109.6	N1—C15—C16	109.28 (19)
C18—N2—H2A	109.6	N1—C15—H15A	109.8
C20—N2—H2A	109.6	C16—C15—H15A	109.8
C2—C1—C6	117.32 (19)	N1—C15—H15B	109.8
C2—C1—C7	122.53 (19)	C16—C15—H15B	109.8
C6—C1—C7	120.10 (19)	H15A—C15—H15B	108.3
C1—C2—C3	121.9 (2)	N2—C16—C15	108.9 (2)
C1—C2—Cl1	119.41 (15)	N2—C16—H16A	109.9
C3—C2—Cl1	118.65 (16)	C15—C16—H16A	109.9
C4—C3—C2	119.4 (2)	N2—C16—H16B	109.9
C4—C3—H3A	120.3	C15—C16—H16B	109.9
C2—C3—H3A	120.3	H16A—C16—H16B	108.3
C3—C4—C5	120.4 (2)	N1—C17—C18	109.5 (2)
C3—C4—H4A	119.8	N1—C17—H17A	109.8
C5—C4—H4A	119.8	C18—C17—H17A	109.8
C4—C5—C6	118.9 (2)	N1—C17—H17B	109.8
C4—C5—H5A	120.6	C18—C17—H17B	109.8
C6—C5—H5A	120.6	H17A—C17—H17B	108.2
C1—C6—C5	122.1 (2)	N2—C18—C17	109.06 (19)
C1—C6—H6A	119.0	N2—C18—H18A	109.9
C5—C6—H6A	119.0	C17—C18—H18A	109.9
O2—C7—O1	124.7 (2)	N2—C18—H18B	109.9
O2—C7—C1	120.2 (2)	C17—C18—H18B	109.9
O1—C7—C1	115.07 (19)	H18A—C18—H18B	108.3
C9—C8—C13	117.1 (2)	N1—C19—C20	109.92 (18)
C9—C8—C14	123.97 (19)	N1—C19—H19A	109.7
C13—C8—C14	118.9 (2)	C20—C19—H19A	109.7
C8—C9—C10	122.1 (2)	N1—C19—H19B	109.7
C8—C9—Cl2	119.51 (16)	C20—C19—H19B	109.7
C10—C9—Cl2	118.36 (18)	H19A—C19—H19B	108.2
C11—C10—C9	119.5 (2)	N2—C20—C19	108.11 (18)
C11—C10—H10A	120.3	N2—C20—H20A	110.1
C9—C10—H10A	120.3	C19—C20—H20A	110.1
C12—C11—C10	119.9 (2)	N2—C20—H20B	110.1
C12—C11—H11A	120.0	C19—C20—H20B	110.1
C10—C11—H11A	120.0	H20A—C20—H20B	108.4
			
C6—C1—C2—C3	1.3 (3)	C11—C12—C13—C8	−0.7 (4)
C7—C1—C2—C3	−176.0 (2)	C9—C8—C13—C12	−0.2 (3)
C6—C1—C2—Cl1	−177.17 (17)	C14—C8—C13—C12	−178.5 (2)
C7—C1—C2—Cl1	5.6 (3)	C9—C8—C14—O4	−65.8 (3)
C1—C2—C3—C4	−0.5 (3)	C13—C8—C14—O4	112.4 (3)
Cl1—C2—C3—C4	177.9 (2)	C9—C8—C14—O3	116.1 (2)
C2—C3—C4—C5	−0.8 (4)	C13—C8—C14—O3	−65.7 (3)
C3—C4—C5—C6	1.2 (4)	C17—N1—C15—C16	−65.6 (3)
C2—C1—C6—C5	−0.8 (4)	C19—N1—C15—C16	54.6 (3)
C7—C1—C6—C5	176.6 (2)	C18—N2—C16—C15	52.6 (3)
C4—C5—C6—C1	−0.4 (4)	C20—N2—C16—C15	−66.6 (3)
C2—C1—C7—O2	75.5 (3)	N1—C15—C16—N2	10.2 (3)
C6—C1—C7—O2	−101.7 (3)	C19—N1—C17—C18	−65.0 (3)
C2—C1—C7—O1	−106.1 (2)	C15—N1—C17—C18	54.4 (3)
C6—C1—C7—O1	76.7 (3)	C16—N2—C18—C17	−63.8 (3)
C13—C8—C9—C10	1.3 (3)	C20—N2—C18—C17	55.4 (3)
C14—C8—C9—C10	179.6 (2)	N1—C17—C18—N2	8.7 (3)
C13—C8—C9—Cl2	179.07 (17)	C17—N1—C19—C20	53.6 (3)
C14—C8—C9—Cl2	−2.7 (3)	C15—N1—C19—C20	−67.1 (3)
C8—C9—C10—C11	−1.5 (4)	C16—N2—C20—C19	54.3 (3)
Cl2—C9—C10—C11	−179.29 (19)	C18—N2—C20—C19	−66.4 (3)
C9—C10—C11—C12	0.5 (4)	N1—C19—C20—N2	10.7 (3)
C10—C11—C12—C13	0.5 (4)		

### Hydrogen-bond geometry (Å, °)

**Table:**

*D*—H···*A*	*D*—H	H···*A*	*D*···*A*	*D*—H···*A*
N1—H1A···O1	0.91	1.65	2.556 (2)	170
N2—H2A···O3	0.91	1.69	2.587 (2)	169
